# Use of Isotretinoin in Rhinoplasty: A Literature Review

**DOI:** 10.7759/cureus.81689

**Published:** 2025-04-04

**Authors:** Sulaiman A Althobaiti, Abdulrahman G Alosaimi, Fahad S Alghamdi, Omar A Alsulami, Manar Alghamdi

**Affiliations:** 1 Otolaryngology and Head and Neck Surgery, King Abdulaziz Specialist Hospital, Taif, SAU; 2 Otolaryngology and Head and Neck Surgery, Ohud Hospital, Medinah, SAU; 3 Otolaryngology and Head and Neck Surgery, Al-Baha University, Al-Baha, SAU; 4 Otolaryngology and Head and Neck Surgery, King Abdulaziz University, Jeddah, SAU; 5 Otolaryngology and Head and Neck Surgery, University of Jeddah, Jeddah, SAU

**Keywords:** isotretinoin, nasal reshaping, rhinoplasty, swelling, thick nasal skin

## Abstract

This literature review explores the role of isotretinoin as an adjunct therapy in rhinoplasty, particularly in patients with thick nasal skin. Thick skin, often associated with excessive sebaceous activity, can obscure surgical refinements and compromise aesthetic outcomes. Isotretinoin, known for its ability to reduce sebaceous gland activity, improve skin elasticity, and regulate keratinization, has been investigated for its potential to enhance postoperative results in rhinoplasty patients. This review synthesizes existing studies, including randomized controlled trials (RCTs), retrospective analysis, and ultrasonographic assessments, to evaluate the effectiveness and safety of isotretinoin in this context.

## Introduction and background

Rhinoplasty is among the most common procedures performed by plastic surgeons. The main indications are to improve facial aesthetics and respiratory function and address aesthetic and functional disorders [1,2]. It is one of the most complex and technically demanding procedures in facial plastic surgery. The quality and thickness of the nasal skin are critical factors influencing rhinoplasty outcomes [3,4]. Patients with thick skin often pose a challenge to surgeons due to the skin’s reduced ability to shrink-wrap over the newly sculpted nasal framework [5]. This can obscure surgical refinements, leading to suboptimal aesthetic results.

To address these challenges, isotretinoin, a vitamin A derivative commonly used in treating acne, has been investigated as an adjunct therapy in rhinoplasty. Isotretinoin is known for its ability to reduce sebaceous gland activity [6], decrease inflammation [7], and improve skin elasticity [8]. It is used for most forms of acne [9,10], rhinophyma, the end stage of acne rosacea [11], and sebaceous hyperplasia [12]. Given these properties, it has been hypothesized that preoperative and/or postoperative administration of isotretinoin may enhance rhinoplasty outcomes by reducing skin thickness and improving skin drape over the nasal framework [13].

This literature review synthesizes the findings of studies examining the effectiveness of isotretinoin as an adjuvant treatment in rhinoplasty for patients with thick nasal skin. It evaluates the impact of isotretinoin on patient satisfaction, surgical outcomes, complications, and long-term results.

## Review

The rationale for the use of isotretinoin in rhinoplasty

The primary challenge in thick-skinned rhinoplasty patients lies in the nature of the skin envelope, which may exhibit increased sebaceous activity and reduced elasticity [14]. These characteristics make it difficult for the skin to conform to the underlying nasal structure after surgical modifications. The thickness of nasal skin is classified using the Fitzpatrick classification system, where types 1 and 2 are considered thin skin, types 3 and 4 are considered intermediate skin, and types 5 and 6 are classified as thick skin. The ideal skin for rhinoplasty is intermediate [3]. Though this classification system directly quantifies the skin color and not the skin thickness, it is still widely used as patients with light skin color have thin skin, whereas those with dark skin have thick skin [15]. The thickness also varies in different parts of the nose and by gender. "In a study by Alharethy et al. evaluating satisfaction in 154 post-rhinoplasty patients, the maximum thickness was found on the upper nose (nasion), followed by the tip and the middle part of the nose (rhinion). There was a significant difference among genders, with higher thickness in males, and this difference reduced as measured from the upper to the lower nose [16]. However, they found that the satisfaction rates after rhinoplasty do not depend on the thickness of the skin. Thin or ultrathin skin shows aggressive scar and contracture; however, nasal tip definition is more easily achieved than in thicker skin [3,17]. The thicker-skinned individuals also have weaker underlying osseocartilaginous structures. This interplay between thicker skin and osseocartilaginous structures exacerbates the loss of definition in cosmetic rhinoplasty due to a reduction in the tip projection. Moreover, these individuals have higher sebaceous glands and produce more inflammation and edema, leading to scar tissue formation, supratip deformity, and unfavorable aesthetic outcomes. Therefore, Cobo et al. classified thick skin into three subtypes for better clinical decisions in these patients. These classify thick-skinned patients based on elasticity, oiliness, and the presence of acne, redness, and pores [18]. The full details are mentioned in Table 1.

**Table 1:** Thick-skin classification Skin thickness classification as per the work done by Cobo et al. [18].

	Thickness	Oiliness	Acne
Type 1	Thick skin with elasticity	Present	No acne
Type 2	Thick skin with no elasticity	Present	Mild to moderate acne, open pores present
Type 3	Thick skin with no elasticity	Present	Moderate to severe acne, open pores present

The first reports of isotretinoin use in rhinoplasty were met with skepticism due to concerns over potential adverse effects, including impaired wound healing, nasal tip deformities, and skin necrosis [19]. However, subsequent studies have provided mixed evidence, highlighting both this approach's possible advantages and limitations. Isotretinoin was approved for the treatment of recalcitrant acne vulgaris in 1982 [20]. Isotretinoin has been widely studied in dermatology for its effects on sebaceous gland suppression, collagen remodeling, and skin thinning. It reduces comedogenesis by decreasing hyperkeratinization [21]. A randomized controlled trial (RCT) by Silveira et al. utilized ultrasonography to objectively measure changes in nasal skin thickness after isotretinoin treatment. In their cohort of 24 patients, the intervention group was found to have a significant reduction in epidermal and dermal thickness at six months after rhinoplasty. These findings support the hypothesis that isotretinoin can alter the skin’s structural properties, potentially improving its adaptability to nasal reshaping [13]. However, the lack of long-term follow-up beyond six months limits the ability to assess whether these changes persist over time. However, these suggest a potential benefit in optimizing rhinoplasty outcomes by improving postoperative skin contraction and reducing excessive swelling.

One of the primary concerns in thick-skinned rhinoplasty patients is prolonged post-operative swelling, which can obscure final surgical results. Studies have suggested that isotretinoin may reduce this swelling by decreasing sebaceous gland activity and associated inflammation. Yahyavi et al. assessed skin oiliness and acne severity postoperatively, finding that isotretinoin significantly reduced oil production in the early months following surgery [22]. However, these effects diminished by 6 to 12 months postoperatively.

While these findings indicate that isotretinoin may enhance early healing, the results must be interpreted with caution. Swelling reduction alone does not necessarily translate into improved long-term surgical outcomes, and other factors such as surgical technique and postoperative care play critical roles.

Dose, duration, and side effects of isotretinoin use

Isotretinoin is given orally and is preferred with food due to its lipophilic nature. The dose varies from 0.3 to 2 mg/kg/day. However, due to its high side effect profile, it is started at the dose of 0.5 mg/kg/day for the initial month and then adjusted to 1 mg/kg/day or more depending on the clinical results. The target is to obtain the accumulated dose of 120 to 150 mg/kg, which showed an improvement rate and fewer relapses by 85% [23,24]. The mean half-life is 20 hours (7 to 39 hours) and is usually given in a twice-daily dosage. Depending on the pathology and severity, the treatment may be given for 15-24 weeks. Although there are a lot of controversies associated with isotretinoin when used with rhinoplasty, studies suggest that low-dose isotretinoin (0.25-0.5 mg/kg/day) administered for at least four months preoperatively may yield the best results in terms of skin thinning and elasticity improvement. Research by Yigit et al. demonstrated that systemic isotretinoin significantly reduced dermal thickness and improved skin elasticity with a minimum duration of a four-month regimen [25]. Per traditional guidelines, it was recommended to wait for 6-12 months for surgery after stopping isotretinoin [3]. Recent studies have shown that even a four-week gap is sufficient to avoid skin healing issues. Ungarelli et al. evaluated 47 studies and investigated important aspects of isotretinoin use [26]. They concluded that isotretinoin does not promote skin healing issues. It is evident from the existing literature that low-dose isotretinoin (10 mg/day) is safe with many procedures, including rhinoplasty [8,27-29].

Some protocols recommend continuing isotretinoin postoperatively for an additional two to six months to maintain its effects on sebum regulation and inflammation control. However, the decision to continue treatment postoperatively should be based on individual patient response and risk assessment. A double-blind RCT by Sazgar et al. [30] observed the effects of postoperative isotretinoin compared to placebo. They started on the 31st day after surgery, thereafter every other day for one month, and further daily for another two months. The patients were evaluated at three months, six months, and one year after surgery. They reported improvement in postoperative edema and cosmetic results during the early months, but the outcome at one year was similar in both groups. This suggests that isotretinoin may accelerate early postoperative improvements, but its long-term impact remains uncertain. In another published study by Allen and Rhee in 2005, three patients were evaluated wherein isotretinoin was started post-rhinoplasty within two years. All patients have nasal tip deformity complications like nasal bossa formation, asymmetry, and prominence of the composite graft. All patients underwent revision surgery for correction, and the authors recommended refraining from isotretinoin use for at least two years after surgery [19].

Despite its potential benefits, the use of isotretinoin in rhinoplasty patients raises concerns. One of the primary apprehensions is its impact on wound healing. Historically, early reports suggested that isotretinoin use was associated with an increased risk of hypertrophic scarring, delayed healing, and keloid formation. These concerns led to recommendations that surgical procedures be postponed for 6 to 12 months following isotretinoin discontinuation [19]. However, more recent studies have challenged these claims, indicating that isotretinoin does not significantly impair wound healing in healthy individuals undergoing dermatological procedures. A systematic review [31] examining isotretinoin’s effects on surgical outcomes found no conclusive evidence linking the drug to increased complication rates, except in cases involving mechanical dermabrasion and fully ablative laser procedures. Additionally, a multicenter study by the Indian Association of Cutaneous Surgeons concluded that isotretinoin does not pose a significant risk in dermatologic surgeries, further supporting its potential safety in rhinoplasty [32]. We have already discussed more studies about this in the previous paragraphs.

Beyond wound healing, isotretinoin is known for its extensive side effects profile, which must be considered when prescribing the drug. Common adverse effects include mucocutaneous dryness, cheilitis, and epistaxis, which are generally manageable with supportive care [14,33]. In a study by Baser et al., these complications were either managed using local application of glycerin or by reducing the dose of isotretinoin to 10 mg on alternate days [14]. More serious but less common side effects include elevated liver enzymes (isotretinoin is metabolized in the liver), hyperlipidemia, and, in rare cases, psychiatric symptoms such as depression and suicidal ideation. Hyperlipidemia was not found concerning in most of the studies and was found to return to normal when the treatment was completed [34]. The most concerning risk is isotretinoin’s teratogenicity. Even a single dose of isotretinoin during pregnancy can lead to severe congenital malformations. This necessitates strict adherence to contraceptive measures and enrollment in pregnancy prevention programs such as the iPLEDGE system in the United States [20].

Patient satisfaction and aesthetic outcomes

There are a few studies that reported improved aesthetic satisfaction among patients who received isotretinoin. In particular, studies by Baser et al. [14] and Pozzi et al. [35] found that patients who received isotretinoin had higher scores on rhinoplasty outcome evaluations (ROEs) and subjective satisfaction measures. Both these studies used ROE as the parameter and found scores >50 in 96% of patients at one year postoperatively in Baser et al. [14] and a mean of 92.12 in the study by Pozzi et al. [35], representing a satisfactory outcome. In Baser et al. [14], 77% of patients reported "marked satisfaction" with the outcomes of the surgery. However, a major limitation was the absence of control groups in some studies, making it difficult to isolate the effects of isotretinoin from the natural healing process.

On the other hand, the study by Sazgar et al. [30], as discussed above, a double-blind RCT, found a significant difference in surgical outcomes utilizing a five-point grading scale (excellent, good, fair, no change, and poor) at three and six months but was similar at one year when compared to placebo. Similar results were reported by Yahyavi et al. [22], where patients reported good outcomes at one and three months, but not at 6 and 12 months when compared to placebo. But when these scores were considered as an average within one year, the treatment group was found to have "higher average satisfaction" rates compared to the placebo.

Silveira et al. [13] used different scoring systems to report outcomes in terms of patient satisfaction. They used the Utrecht questionnaire, which is based on visual analog scale (VAS) questions for reporting mean satisfaction and the Likert scale to report a concern with the appearance of the nose. However, no significant difference was found between the treatment group and the control group. Therefore, with the above studies, it is evident that the long-term postoperative benefit of isotretinoin is still not clear in terms of patient satisfaction and surgical outcomes.

Future direction in research

While current evidence suggests the potential benefits of isotretinoin in rhinoplasty, several unanswered questions remain. Future research should focus on large-scale RCTs with standardized treatment protocols to establish definitive guidelines. Key areas of investigation should include long-term efficacy, optimal treatment duration, and comparative studies. Head-to-head comparisons between isotretinoin and other skin-modifying therapies (e.g., topical retinoids, laser treatments) could provide insight into the most effective strategies for optimizing skin quality in rhinoplasty patients.

## Conclusions

The integration of isotretinoin into rhinoplasty treatment plans for patients with thick skin offers a promising approach to improving surgical outcomes. By reducing sebaceous activity, enhancing skin contraction, and minimizing postoperative swelling, isotretinoin has the potential to address some of the challenges associated with thick-skinned rhinoplasty cases. However, its use must be carefully tailored to individual patient needs, with consideration given to safety, monitoring, and ethical implications. While recent evidence supports the safe use of isotretinoin in rhinoplasty patients, further research is warranted to refine treatment protocols and establish best practices. Until more definitive guidelines are available, isotretinoin should be prescribed with caution, ensuring that its benefits outweigh the potential risks for each patient.

## References

[1] International Society of Aesthetic Plastic Surgery (2017). International Survey on Aesthetic/Cosmetic Procedures Performed in 2017.

[2] (2017). American Society of Plastic Surgeons. https://www.plasticsurgery.org/documents/News/Statistics/2017/plastic-surgery-statistics-full-report-2017.pdf.

[3] Guyuron B, Lee M (2017). An effective algorithm for management of noses with thick skin. Aesthetic Plast Surg.

[4] Vahidi N, Wang L, Peng GL, Nassif P, Azizzadeh B (2023). Rhinoplasty for thick-skinned noses: a systematic review. Aesthetic Plast Surg.

[5] Rohrich RJ, Ahmad J (2016). A practical approach to rhinoplasty. Plast Reconstr Surg.

[6] Zouboulis CC (2006). Isotretinoin revisited: pluripotent effects on human sebaceous gland cells. J Invest Dermatol.

[7] Esen M (2024). Effect of isotretinoin treatment on inflammatory and hematological parameters in patients with acne vulgaris. Cutan Ocul Toxicol.

[8] Hernandez-Perez E, Khawaja HA, Alvarez TY (2000). Oral isotretinoin as part of the treatment of cutaneous aging. Dermatol Surg.

[9] Orozco B, CampoME CampoME, Anaya LA (2011). Colombian guidelines for the management of acne: an evidence-based review by the Colombian acne study group (Article in Spanish). Rev Assoc Colomb Dermatol.

[10] Dréno B, Bettoli V, Ochsendorf F (2006). An expert view on the treatment of acne with systemic antibiotics and/or oral isotretinoin in the light of the new European recommendations. Eur J Dermatol.

[11] Husein-ElAhmed H, Armijo-Lozano R (2013). Management of severe rhinophyma with sculpting surgical decortication. Aesthetic Plast Surg.

[12] Tagliolatto S, Santos Neto Ode O, Alchorne MM, Enokihara MY (2015). Sebaceous hyperplasia: systemic treatment with isotretinoin. An Bras Dermatol.

[13] Silveira CS, Azulay-Abulafia L, Barcaui EO, Silva MM, Roxo AC (2024). Analysis of the use of isotretinoin as an adjuvant in rhinoplasty. Int J Dermatol.

[14] Baser B, Dev S, Mukhopadhyay S (2024). A comprehensive approach to rhinoplasty for thick-skinned patients. J Laryngol Otol.

[15] Ly BC, Dyer EB, Feig JL, Chien AL, Del Bino S (2020). Research techniques made simple: cutaneous colorimetry: a reliable technique for objective skin color measurement. J Invest Dermatol.

[16] Alharethy S, Mousa A, Alharbi A, Aldrees T, AlQaryan S, Ju Jang Y (2018). Does skin thickness affect satisfaction post rhinoplasty? Middle Eastern population as an example. Saudi Med J.

[17] Cho GS, Kim JH, Yeo NK, Kim SH, Jang YJ (2011). Nasal skin thickness measured using computed tomography and its effect on tip surgery outcomes. Otolaryngol Head Neck Surg.

[18] Cobo R, Camacho JG, Orrego J (2018). Integrated management of the thick-skinned rhinoplasty patient. Facial Plast Surg.

[19] Allen BC, Rhee JS (2005). Complications associated with isotretinoin use after rhinoplasty. Aesthetic Plast Surg.

[20] Zaenglein AL, Pathy AL, Schlosser BJ (2016). Guidelines of care for the management of acne vulgaris. J Am Acad Dermatol.

[21] Dalziel K, Barton S, Marks R (1987). The effects of isotretinoin on follicular and sebaceous gland differentiation. Br J Dermatol.

[22] Yahyavi S, Jahandideh H, Izadi M, Paknejad H, Kordbache N, Taherzade S (2020). Analysis of the effects of isotretinoin on rhinoplasty patients. Aesthet Surg J.

[23] Truchuelo MT, Jiménez N, Mavura D, Jaén P (2015). Assessment of the efficacy and safety of a combination of 2 topical retinoids (RetinSphere) in maintaining post-treatment response of acne to oral isotretinoin. Actas Dermosifiliogr.

[24] Zane LT, Leyden WA, Marqueling AL, Manos MM (2006). A population-based analysis of laboratory abnormalities during isotretinoin therapy for acne vulgaris. Arch Dermatol.

[25] Yigit E, Rakici IT, Seden N, Manav V, Kaygisiz I, Yigit O (2022). The impact of isotretinoin therapy on the nasal skin thickness and elasticity: an ultrasonography and elastography based assessment in relation to dose and duration of therapy. Aesthetic Plast Surg.

[26] Ungarelli LF, Hetem CM, Farina Junior JA (2016). Is it safe to operate on patients taking isotretinoin?. Aesthetic Plast Surg.

[27] Heppt MV, Kirchberger MC, Ruzicka T, Berking C, Heppt WJ (2018). Indications and use of isotretinoin in facial plastic surgery. Facial Plast Surg.

[28] Yoon JH, Park EJ, Kwon IH (2014). Concomitant use of an infrared fractional laser with low-dose isotretinoin for the treatment of acne and acne scars. J Dermatolog Treat.

[29] Xia J, Hu G, Hu D, Geng S, Zeng W (2018). Concomitant use of 1,550-nm nonablative fractional laser with low-dose isotretinoin for the treatment of acne vulgaris in Asian patients: a randomized split-face controlled study. Dermatol Surg.

[30] Sazgar AA, Majlesi A, Shooshtari S, Sadeghi M, Sazgar AK, Amali A (2019). Oral isotretinoin in the treatment of postoperative edema in thick-skinned rhinoplasty: a randomized placebo-controlled clinical trial. Aesthetic Plast Surg.

[31] Mysore V, Omprakash HM, Khatri GN (2019). Isotretinoin and dermatosurgical procedures. Indian J Dermatol Venereol Leprol.

[32] Mahadevappa OH, Mysore V, Viswanath V (2016). Surgical outcome in patients taking concomitant or recent intake of oral isotretinoin: a multicentric study-ISO-AIMS study. J Cutan Aesthet Surg.

[33] Afzalzadeh MR, Alizadeh A (2023). Oral isotretinoin treatment in rhinoplasty: a review. World J Plast Surg.

[34] Kandathil CK, Rossi-Meyer M, Saltychev M, Most SP (2025). Effectiveness of isotretinoin administration in rhinoplasty: a systematic review. Facial Plast Surg Aesthet Med.

[35] Pozzi M, Fàdel C, Bolletta A, Cuomo R, Roxo CW (2023). Ethnic rhinoplasty: preliminary results of our technique in the pursuit of the harmonious nose. J Plast Reconstr Aesthet Surg.

